# Ionic liquid containing high-density polyethylene supported tungstate: a novel, efficient, and highly recoverable catalyst

**DOI:** 10.3389/fchem.2024.1346108

**Published:** 2024-02-29

**Authors:** Farideh Mousavi, Dawood Elhamifar, Shiva Kargar, Davar Elhamifar

**Affiliations:** ^1^ Department of Chemistry, Yasouj University, Yasouj, Iran; ^2^ Department of Chemical Engineering, Iran University of Science and Technology (IUST), Tehran, Iran

**Keywords:** polyethylene, tetrahydrobenzo[a]xanthen-11-ones, ionic liquid, nanocomposite, catalyst

## Abstract

Synthesis and catalytic application of polymeric-based nanocomposites are important subjects among researchers due to their high lipophilicity as well as high chemical and mechanical stability. In the present work, a novel nanocomposite material involving ionic liquid and high-density polyethylene supported tungstate (PE/IL-WO_4_^=^) is synthesized, characterized and its catalytic application is investigated. The coacervation method was used to incorporate 1-methyl-3-octylimidazolium bromide ([MOIm] [Br]) ionic liquid in high-density polyethylene, resulting in a PE/IL composite. Subsequently, tungstate was anchored on PE/IL to give PE/IL-WO_4_^=^ catalyst. The PXRD, FT-IR, EDX, TGA, and SEM analyses were used to characterize the PE/IL-WO_4_^=^ composite. This material demonstrated high catalytic efficiency in the synthesis of bioactive tetrahydrobenzo[a]xanthen-11-ones under green conditions. The recoverability and leching tests were performed to investigate the stability and durability of the designed PE/IL-WO_4_^=^ catalyst under applied conditions.

## 1 Introduction

Nowadays, one of the most important challenges in organic chemistry is the synthesis and development of chemically stable, highly efficient, and recoverable catalysts (Oozeerally et al., 2018; Jiang et al., 2020; Chen et al., 2023; Jain et al., 2023). To improve the activity and recoverability of homogeneous catalysts, a wide variety of solid materials have been developed as support (Franco et al., 2020; Saito and Kobayashi, 2020; Das et al., 2021). Some attractive supports that have been used for the heterogenization of the catalysts are molecular sieves (Rimaz et al., 2022; Gao et al., 2023), magnetic nanoparticles (Xie and Wang, 2021; Xie and Li, 2023), montmorillonite (Chellapandi and Madhumitha, 2022; Liu et al., 2023), commercial silica (Peron et al., 2021; Chandrashekhar et al., 2022), two-dimensional manganese dioxide (MnO_2_) (Das et al., 2020), and metal-organic frameworks (MOFs) (Goetjen et al., 2020; Lin et al., 2022). In particular, polymeric materials have drawn a lot of interest as potential catalytic support due to their high stability, easy synthesis and functionalization, and strong corrosion resistance (Gokmen and Du Prez, 2012; Shi et al., 2013; Aziz and Islam, 2018; Jiang et al., 2021; Jiang et al., 2022). Moreover, functionalized polymers are extensively employed in various industries such as packaging (Wan et al., 2020), transportation (Sarkar et al., 2019), biomedical engineering (Szymczyk-Ziółkowska et al., 2020), sporting goods (Sharma et al., 2020), electronics (Zhang et al., 2020), energy storage (Zhang et al., 2021), and water treatment (Khodakarami and Bagheri, 2021; Das et al., 2023). Some recently developed catalysts in this matter are Pd/PVPy (Fusini et al., 2020), Poly-NHC-2–Pd^2+^ (Xu et al., 2015), Au-NWs@Pd@PEI (Xue et al., 2018), PS-TRIP (Clot-Almenara et al., 2016), and Pd@PANI (Wang et al., 2019), PEEK-TBD (Shi et al., 2023). Among different polymers, polyethylene (PE) is widely regarded as a highly versatile material owing to its exceptional workability, chemical inertness, affordability, high resistance to elevated temperatures, and extensive compatibility with various processing techniques. Therefore, PE is a promising candidate for the immobilization of homogeneous catalysts (Pribyl et al., 2019; Mohebbi and Farajzadeh, 2020; Kargar et al., 2022). Different studies such as LDPE-supported ZVI (Mossmann et al., 2019), PEt@Zn/IL (Zaki et al., 2021), and PEolig-NHC-Ru (Hobbs et al., 2011) have been recently reported in this regard. However, the catalyst leaching and inaccessibility to the active catalytic sites are limitations of the most of the aforementioned systems. Therefore, the design and development of an effective and robust PE-supported catalytic system is an important objective in this matter.

On the other hand, ionic liquids (ILs) are extremely important compounds with a wide range of potential applications because of their hydrophobicity that can be adjusted, excellent solubility with numerous compounds, and negligible vapor pressure. These compounds have a high ability to stabilize polar and charged catalysts due to their inherent ionic nature. For instance, imidazolium-based ionic liquids are highly effective in stabilizing transition metal complexes, thereby enhancing their catalytic activities (Ni and Headley, 2010; Karimi et al., 2018; Gao et al., 2021; Taheri et al., 2023). Thus, combining imidazolium-based ILs with polyethylene supports provides remarkable properties, including high activity, selectivity, and reproducibility.

The synthesis of tetrahydrobenzo[a]xanthen-11-ones has also gained significant attention from chemists due to their notable biological features such as antibacterial, antiviral, anti-tumor, and antimalaria activities (Chibale et al., 2003; Nandi et al., 2011; Mohammadi et al., 2014; Soliman and Khatab, 2018). Tetrahydrobenzo[a]xanthen-11-ones are synthesized via condensation of aromatic aldehydes, 1,3-dicarbonyl compounds, and β-naphthols in the presence of acid catalysts such as NaHSO_4_-SiO_2_ (Das et al., 2007), p-toluenesulfonic acid (Janardhan et al., 2012), InCl_3_/P_2_O_5_ (Nandi et al., 2009), Caro’s acid-silica gel (Karimi et al., 2010), ruthenium chloride (Tabatabaeian et al., 2011), MSNBA-5 (Ray et al., 2014) and phenylboronic acid (Goswami et al., 2011). There have been many reported methods to prepare tetrahydrobenzo[a]xanthen-11-ones (Oskooie et al., 2011; Mirjalili et al., 2012; Mondal et al., 2012; Moosavi-Zare et al., 2013; Bahrami et al., 2014). However, these have several drawbacks, including high catalyst loading, low yields of the desired products and the use of pricey ligands, time-consuming workups, challenging product and catalyst separation, and the use of dangerous solvents. Therefore, it is very important to develop an environmentally benign and highly efficient method for the synthesis of tetrahydrobenzo[a]xanthen-11-ones.

In view of the above and considering the advantages of ionic liquid/polymer composites, this study presents the synthesis of a newly developed nanocomposite consisting of polyethylene and an ionic liquid, which serves as a support for tungstate (PE/IL-WO_4_^=^). Furthermore, the catalytic efficacy of this nanocomposite in the environmentally friendly synthesis of tetrahydrobenzo[a]xanthen-11-ones is investigated.

## 2 Experimental section

### 2.1 Synthesis of PE/IL

The synthesis of the [MOIm] [Br] ionic liquid was done by using a previously reported procedure (Kargar and Elhamifar, 2020). The PE/IL was subsequently synthesized using the coacervation method as described below. In the first step, 1 g of high-density polyethylene (PE) was dissolved in 15 mL of xylene at reflux temperature for 30 min. Afterward, IL (0.4 g) was added to the obtained mixture, and it was heated to reflux for 2 h. Next, the resulting mixture was precipitated in methanol at 4°C. The product was washed completely with MeOH, dried at 70°C for 6 h, and denoted as PE/IL.

### 2.2 Synthesis of PE/IL-WO_4_^=^ catalyst

To do this, 1 g of PE/IL was added to DMSO and thoroughly dispersed under ultrasonic irradiation for 30 min. Then 0.30 g (0.82 mmol) of Na_2_WO_4_.4H_2_O was added, and the resulting mixture was stirred at room temperature for 24 h. After filtration, complete washing with EtOH, and drying at 70°C for 5 h, the PE/IL-WO_4_^=^ product was obtained.

### 2.3 Synthesis of tetrahydrobenzo[a]xanthen-11-ones in the presence of PE/IL-WO_4_^=^ catalyst

To do this, 0.10 mol% of PE/IL-WO_4_^=^ catalyst (based on the amount of W) was added to a mixture of aldehyde (1 mmol), 2-naphthol (1 mmol), dimedone (1 mmol), and ethanol (5 mL). This combination was stirred under reflux conditions. TLC was utilized to monitor the progress of the reaction. After the reaction was finished, the catalyst was removed via filtration, and the pure products were obtained by recrystallizing the residue in EtOH.

### 2.4 IR, ^1^H NMR and ^13^C NMR data of tetrahydrobenzo[a]xanthen-11-ones

#### 2.4.1 9,9-Dimethyl-12-phenyl-8,9,10,12-tetrahydrobenzo[a]xanthen-11-one

White solid; M. P.: 151°C–152°C. FT-IR (KBr, cm^−1^): 3053 (=C–H, stretching vibration sp^2^), 2956 (C–H, stretching vibration sp^3^), 1648 (C=O, stretching vibration), 1619 (C=C, stretching vibration sp^2^), 1593, 1468 (C=C, Ar stretching vibration sp^2^), 1230 (C–O, stretching vibration). ^1^H-NMR (400 MHz, DMSO): δ (ppm) 0.96 (s, 3H), 1.12 (s, 3H), 2.25 (d, 1H, *J* = 16 Hz), 2.30 (d, 1H, *J* = 16.3 Hz), 2.56 (s, 2H), 5.70 (s, 1H), 7.07 (t, 1H, *J* = 7.5 Hz), 7.18 (t, 2H, *J* = 8 Hz), 7.31–7.45 (m, 5H), 7.77 (d, 1H, *J* = 8.3 Hz), 7.79 (d, 1H, *J *= 6.3 Hz), 8.02 (d, 1H *J *= 8.3 Hz). ^13^C-NMR (100 MHz, DMSO) δ (ppm) 28.3, 33.0, 33.9, 40.7, 51.1, 117.4, 119.5, 124.1, 125.4, 127.1, 127.7, 128.1, 128.3, 129.0, 129.2, 130.4, 132.8, 142.4, 153.9, 164.4, 197.7.

#### 2.4.2 9,9-Dimethyl-12-(4-chlorophenyl)-8,9,10,12-tetrahydrobenzo[a]xanthen-11-one

White solid; M. P.: 181°C–183 °C. FT-IR (KBr, cm^−1^): 3068 (=C–H, stretching vibration sp^2^), 2958 (C–H, stretching vibration sp^3^), 1652 (C=O, stretching vibration), 1621 (C=C, stretching vibration sp^2^), 1594, 1479 (C=C, Ar stretching vibration sp^2^), 1225 (C–O, stretching vibration). ^1^H-NMR (400 MHz, DMSO): δ (ppm) 0.97 (s, 3H), 1.08 (s, 3H), 2.28 (d, 1H, *J* = 16.5 Hz), 2.35 (d, 1H, J = 16.1 Hz), 2.64 (s, 2H), 5.73 (s, 1H), 7.36 (d, 1H, J = 9.1), 7.38–7.48 (m, 2H), 7.54 (d, 2H, *J* = 8.9 Hz), 7.84–7.87 (m, 3H), 8.10 (d, 2H, *J *= 8.5 Hz). ^13^C-NMR (100 MHz, DMSO) δ (ppm) 28.3, 33.5, 34.1, 40.7, 51.6, 118.1, 120.0, 123.9, 124.3, 125.6, 127.7, 128.2, 129.3, 129.4, 130.7, 132.8, 147.2, 147.5, 154.3, 164.2, 197.5.

## 3 Results and discussion

**FIGURE 1:**
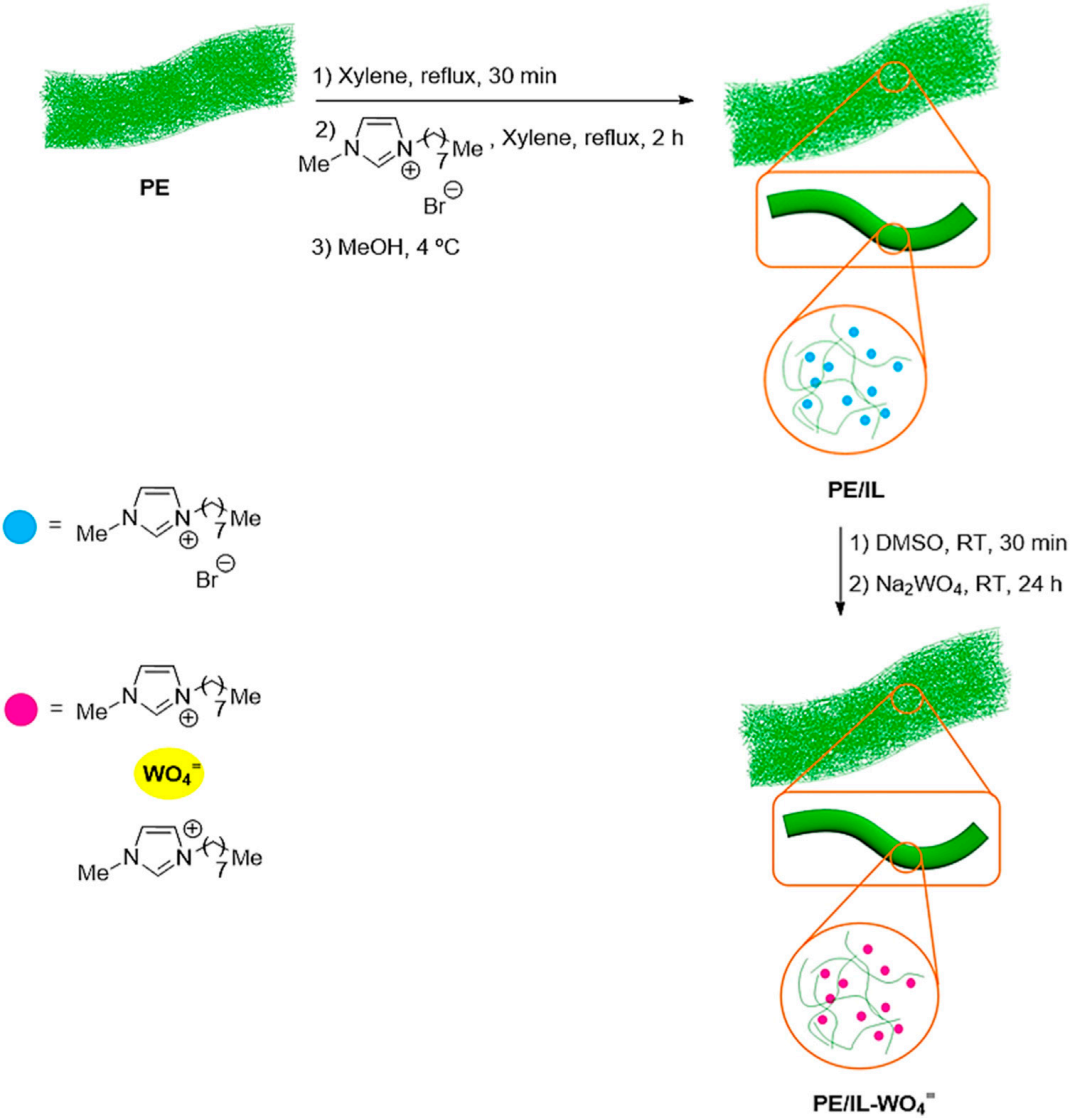
Preparation of PE/IL-WO_4_^=^.

Figure 1 illustrates the preparation method for PE/IL-WO_4_^=^. Initially, the coacervation technique was used to immobilize the [MOIm] [Br] into/onto polyethylene to create PE/IL. To synthesize the PE/IL-WO_4_^=^ composite, the PE/IL material was subsequently treated with Na_2_WO_4_.

**FIGURE 2:**
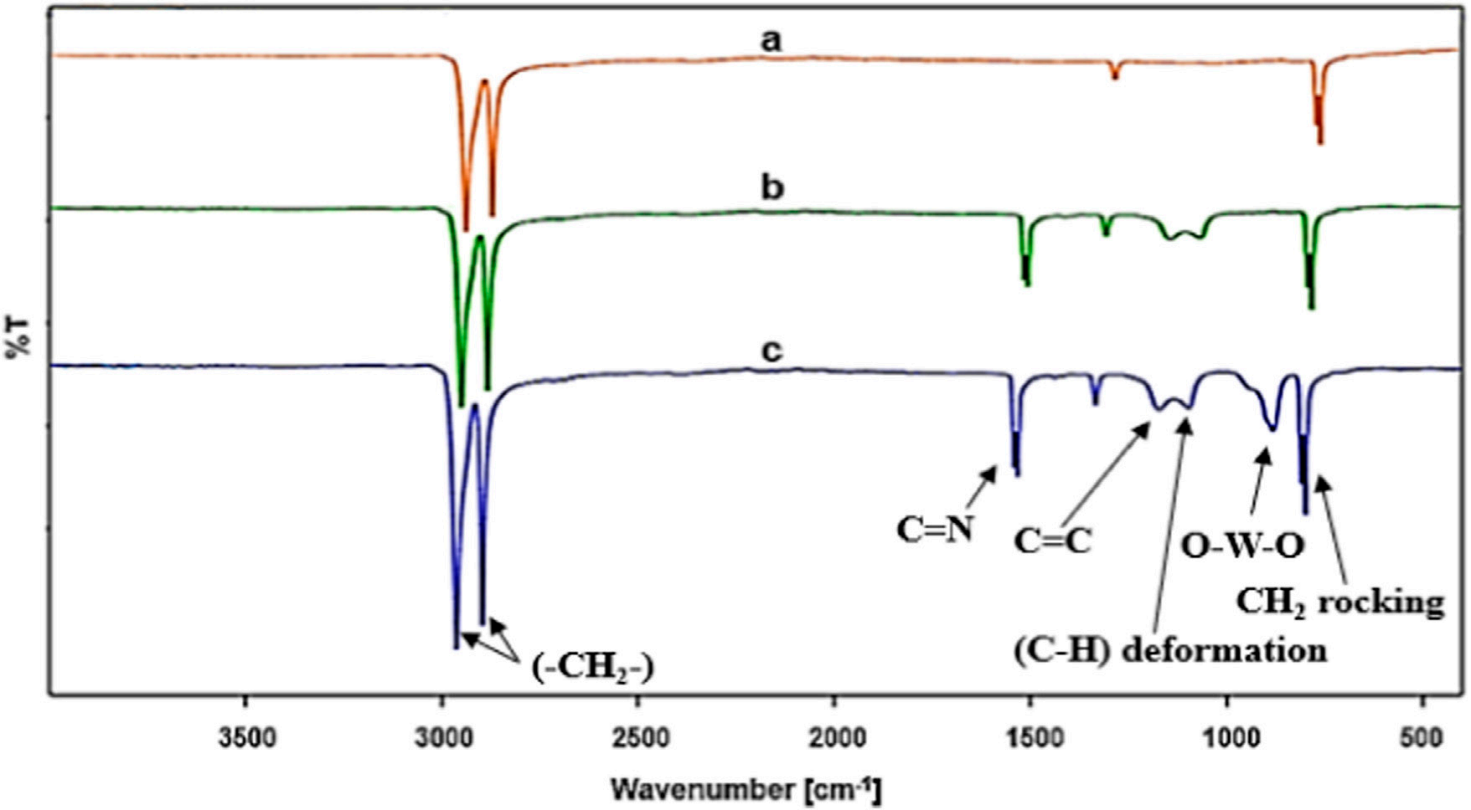
FT-IR of **(A)** PE, **(B)** PE/IL and **(C)** PE/IL-WO_4_^=^.

FT-IR analysis was used to confirm the structure of all prepared materials. Figure 2A shows the characteristic peaks of PE, including the methylene (-CH_2_-) groups stretching vibration at 2920 and 2819 cm^−1^, the C-H deformation at 1453 cm^−1^, and the CH_2_ rocking stretching vibration at 715 cm^−1^. The peaks that appeared at 1635 and 1530 cm^−1^ are attributed to the C=N and C=C bonds of ionic liquid moieties, respectively, confirming the successful incorporation/immobilization of ILs into/onto the polymer framework (Figures 2B, C) (Kargar and Elhamifar, 2020). The band of O-W-O bonds appeared at 828 cm^−1^ proving the successful immobilization of WO_4_^=^ on PE/IL composite (Dkhilalli et al., 2018) (Figure 2C).

**FIGURE 3:**
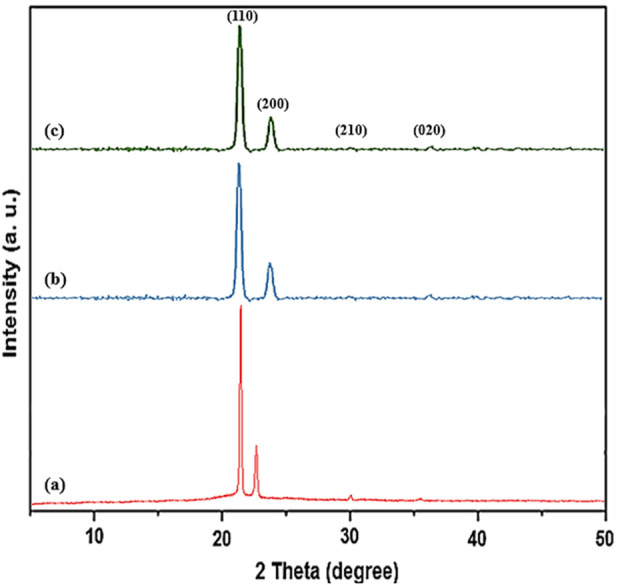
PXRD pattern of **(A)** PE, **(B)** PE/IL, and **(C)** PE/IL-WO_4_^=^ catalyst.

The PXRD of PE, PE/IL, and PE/IL-WO_4_^=^ are shown in Figure 3. As seen, all samples exhibit the typical orthorhombic unit cell structure with the two crystal planes of (110) and (200) at angles of 21.6° and 24.0°, respectively. Additionally, the relatively low-intensity peaks at 2θ of 30.2° and 36.5°, are attributed to the (210) and (020) crystal planes, respectively. These findings are in good agreement with the PXRD pattern of high-density polyethylene (Inci and Wagener, 2011; Chouit et al., 2014), proving that the crystalline structure of PE is maintained throughout the modification procedure. This indicates that the incorporation of IL in the polymer matrix did not affect its original crystalline structure.

**FIGURE 4:**
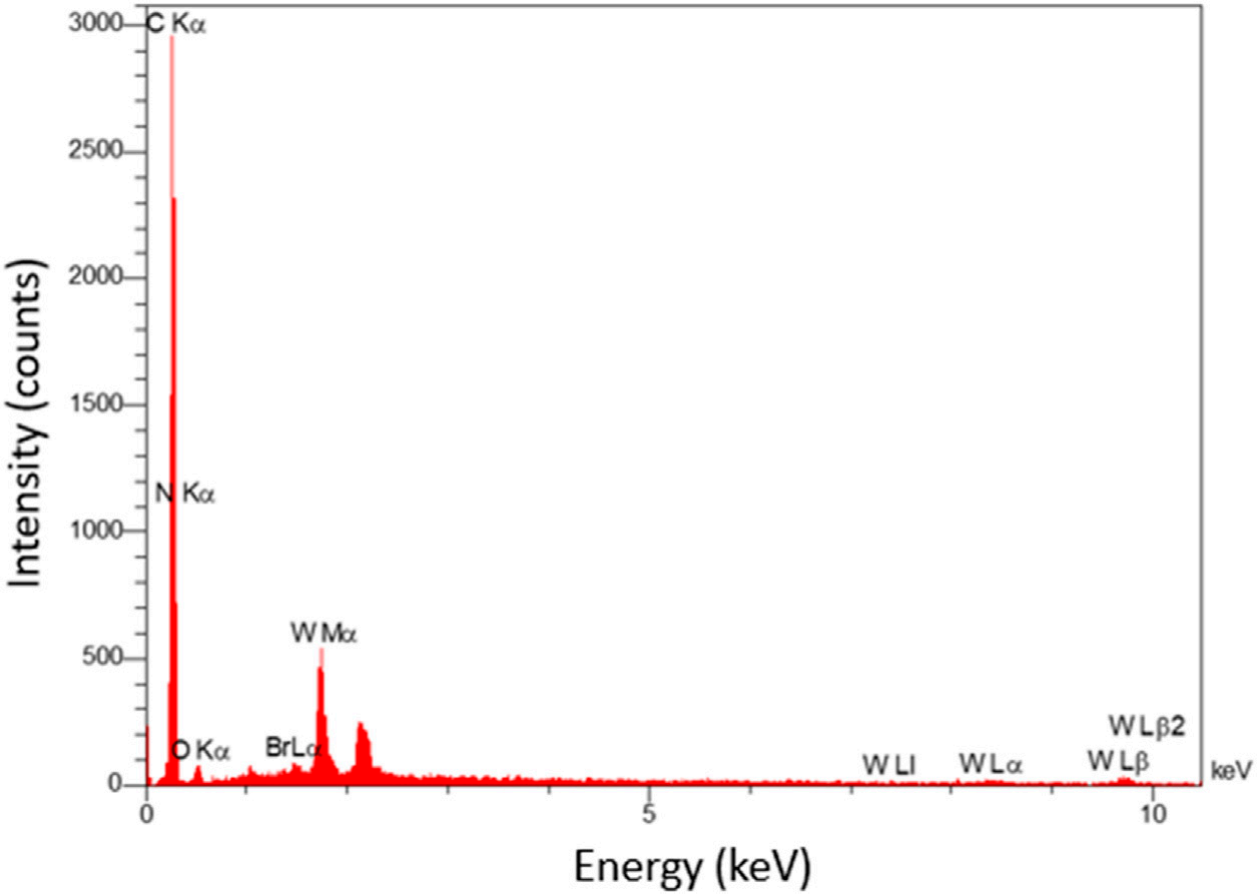
EDX of PE/IL-WO_4_^=^.

Moreover, from the EDX analysis, the successful incorporation/immobilization of IL-WO_4_^=^ complex into/onto the PE network was confirmed by the presence of C, N, O, Br, and W elements (Figure 4), which is in accordance with the FT-IR results.

**FIGURE 5:**
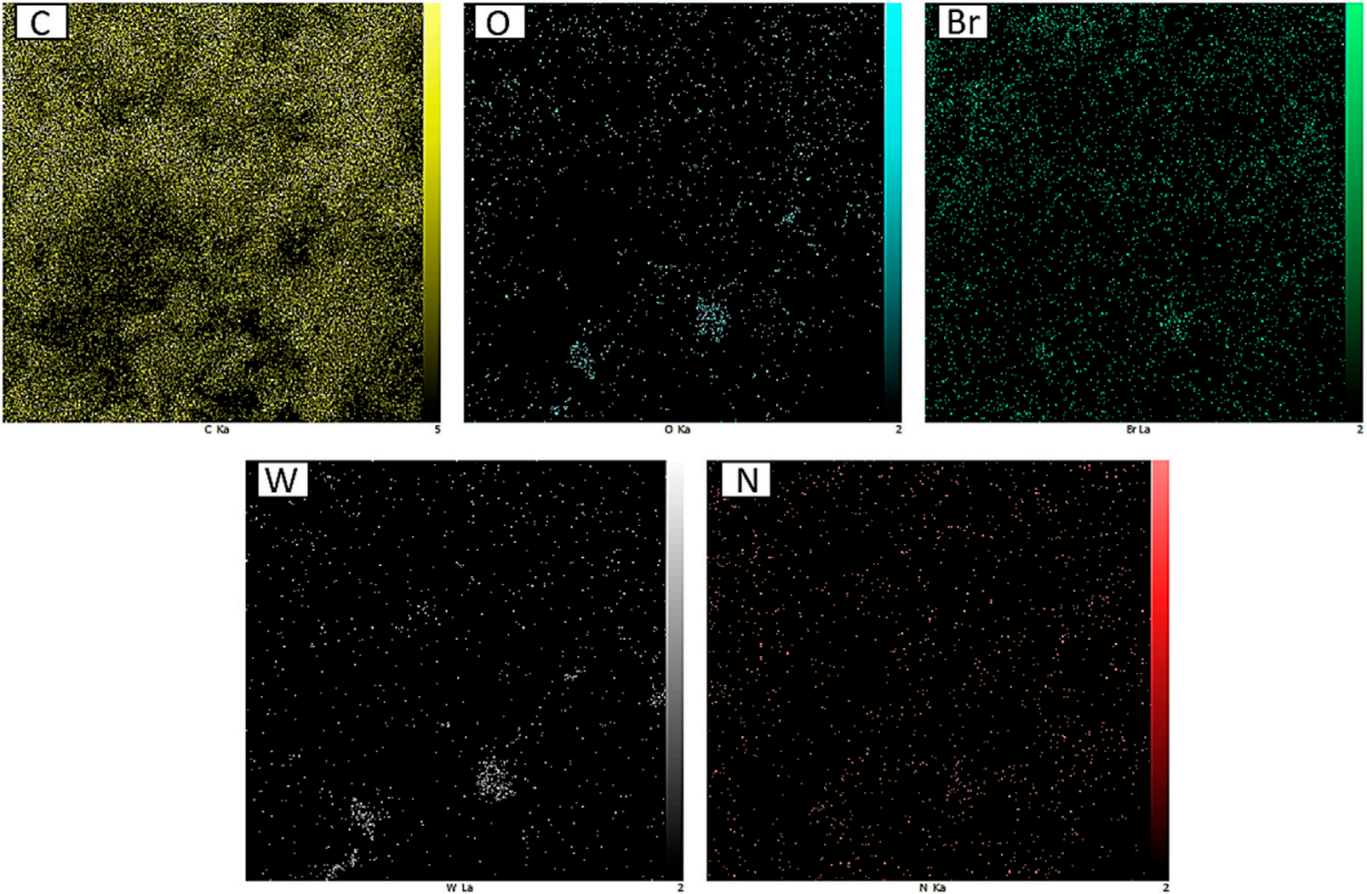
EDX mapping of PE/IL-WO_4_^=^.

In addition, the distribution of the above-mentioned elements was studied by using the EDX mapping analysis (Figure 5), indicating a uniform distribution for all elements throughout the material framework.

**FIGURE 6:**
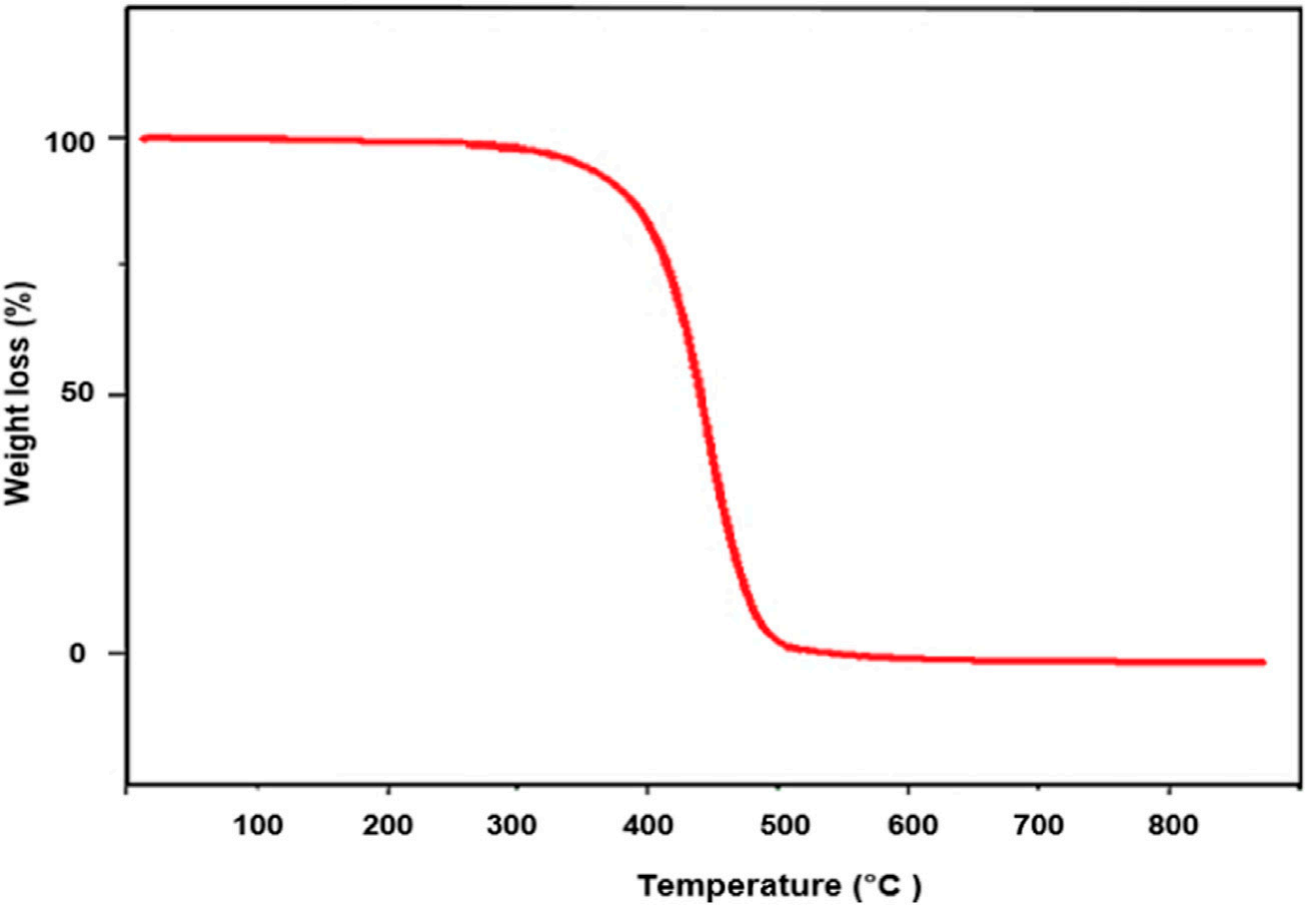
TGA of the PE/IL-WO_4_^=^ catalyst.

TG analysis was performed to determine the thermal stability of PE/IL-WO_4_^=^ composite. The TG curve of the designed catalyst showed two weight losses (Figure 6). The first weight loss, approximately 2%, occurred below 190°C, and resulted from alcoholic solvents and water evaporation. The main weight loss (94%), observed between 400°C and 510°C, is attributed to the decomposition of polyethylene and ionic liquid moieties. These findings demonstrate that the PE/IL-WO_4_^=^ composite is very thermally stable.

**FIGURE 7:**
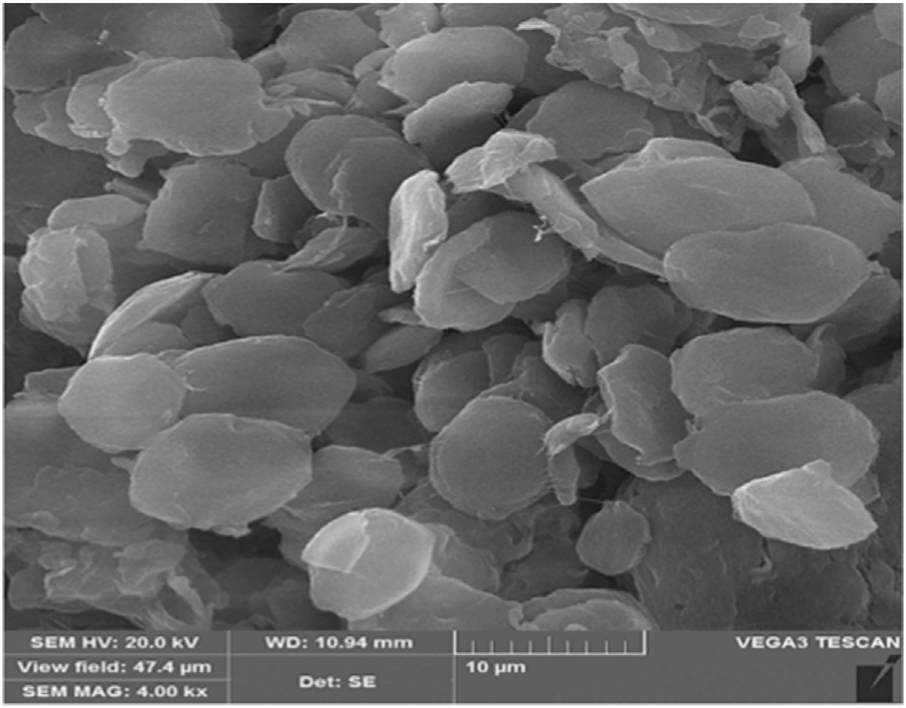
SEM image of the PE/IL-WO_4_^=^ catalyst.

SEM analysis indicates that the PE/IL-WO_4_^=^ particles have a flower-like morphology with uniform size distribution (Figure 7).

After PE/IL-WO_4_^=^ was successfully characterized, its catalytic activity was evaluated in the synthesis of tetrahydrobenzo[a]xanthen-11-ones. For this, the condensation between aldehyde (1 mmol), 2-naphthol (1 mmol), and dimedone was considered as a reaction model. To achieve the optimal conditions, the effect of catalyst loadings, solvents, and reaction temperature were investigated (Table 1). It was found that among different solvents of toluene, ethanol, and water, in EtOH the highest product yield is obtained (Table 1, entries 1–3). The amount of catalyst had an impact on the reaction as well, and the highest yield resulted from using 0.10 mol% of PE/IL-WO_4_^=^ (Table 1, entry 3). The study also demonstrated that the rate of the reaction is affected by temperature, in which the best result was obtained at 78°C (Table 1, entries 6, 7). Accordingly, the use of 0.10 mol% of the PE/IL-WO_4_^=^ catalyst and EtOH solvent under reflux conditions (78°C) was selected as the optimum conditions. The activity of PE/IL and PE materials was subsequently compared to that of PE/IL-WO_4_^=^. No conversion was observed with these W-free materials, confirming that the reaction is actually catalyzed via supported tungsten species (Table 1, entry 3 *versus* entries 9 and 10). In addition, to elucidate the role of the imidazolium-based IL, the reaction was carried out in the presence of the unsupported WO_4_^=^ (Table 1, entry 11). Attractively, this latter case showed a significantly lower catalytic activity compared to the PE/IL-WO_4_^=^. These observations clearly indicate that the ionic liquid moieties prevent the aggregation of WO_4_^=^ species and therefore improve their stability and catalytic activity under applied conditions (Wang et al., 2016).

**TABLE 1:** Effect of catalyst loading, temperature, and solvents in the preparation of tetrahydrobenzo [a]xanthen-11-ones.

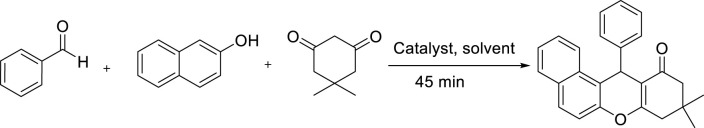					
Entry	Solvent	Catalyst	Catalyst loading (mol%)	Temperature (°C)	Yield (%)	
1	Toluene	PE/IL-WO_4_^=^	0.10	78	58	
2	H_2_O	PE/IL-WO_4_^=^	0.10	78	78	
3	EtOH	PE/IL-WO_4_^=^	0.10	78	93	
4	EtOH	PE/IL-WO_4_^=^	0.05	78	77	
5	EtOH	PE/IL-WO_4_^=^	0.15	78	94	
6	EtOH	PE/IL-WO_4_^=^	0.10	40	52	
7	EtOH	PE/IL-WO_4_^=^	0.10	RT	31	
8	EtOH	-	-	78	-	
9	EtOH	PE/IL	0.01 g	78	12	
10	EtOH	PE	0.01 g	78	-	
11	EtOH	WO4=	0.10	78	75	

Following the optimization of the experimental conditions (Table 1, entry 3, a range of aldehydes were employed as substrate. The tetrahydrobenzo[a]xanthen-11-ones were obtained in high yields using all types of aldehydes, as indicated in Table 2. It is noteworthy that the influence of electronic characteristics and substituent positions on this process was negligible, and the PE/IL-WO_4_^=^ catalyst can catalyze this procedure effectively.

**TABLE 2:** Synthesis of the tetrahydrobenzo [a]xanthen-11-ones catalyzed by PE/IL-WO_4_^=^.

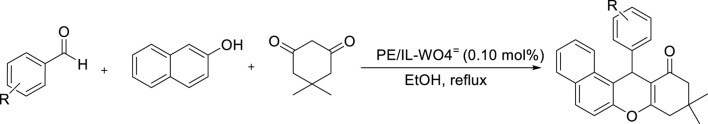					
Entry	Aldehyde	Time (min)	Yield (%)	Found M. P	Reported M. P
1	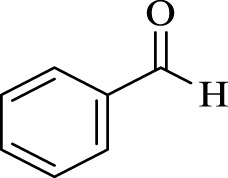	45	93	151–152	151–153 (Nandi et al., 2009)
2	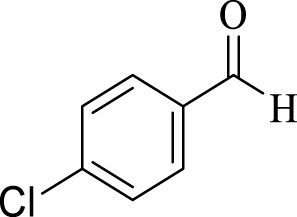	35	95	181–183	180–182 (Nandi et al., 2009)
3	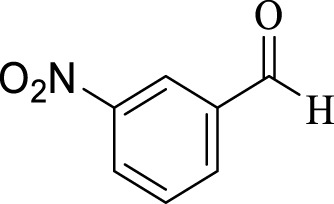	30	96	167–169	166–167 (Zhang et al., 2010)
4	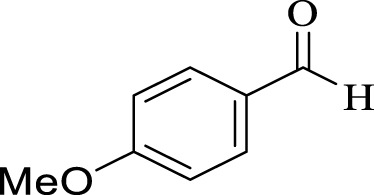	35	87	203–205	201–203 (Khazaei et al., 2012)
5	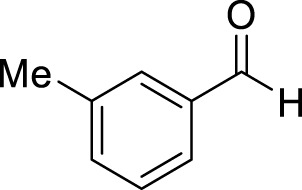	30	84	179–181	178–180 (Khazaei et al., 2012)

**FIGURE 8:**
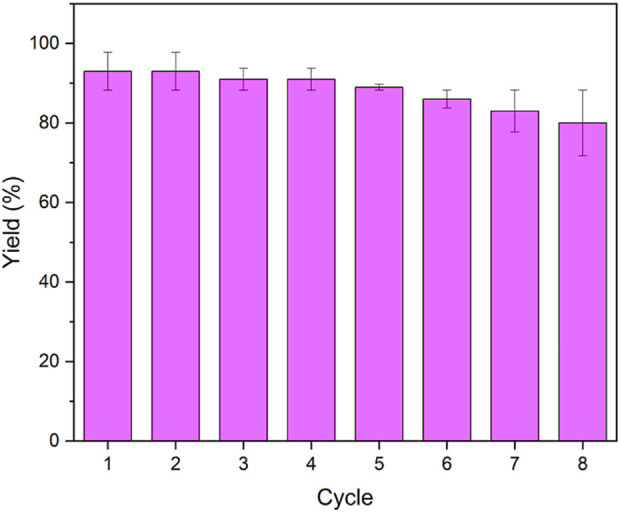
The recoverability and reusability of the PE/IL-WO_4_^=^ catalyst.

In the next study, the recoverability and reusability of PE/IL-WO_4_^=^ were investigated under optimal conditions. For this, the condensation between benzaldehyde, dimedone, and 2-naphthol was used as a test model. After completing the reaction, the PE/IL-WO_4_^=^ catalyst was separated via filtration and reused under identical conditions as the initial run. These steps were repeated and it was found that PE/IL-WO_4_^=^ can be recovered and reapplied at least seven times without losing its activity (Figure 8).

**FIGURE 9:**
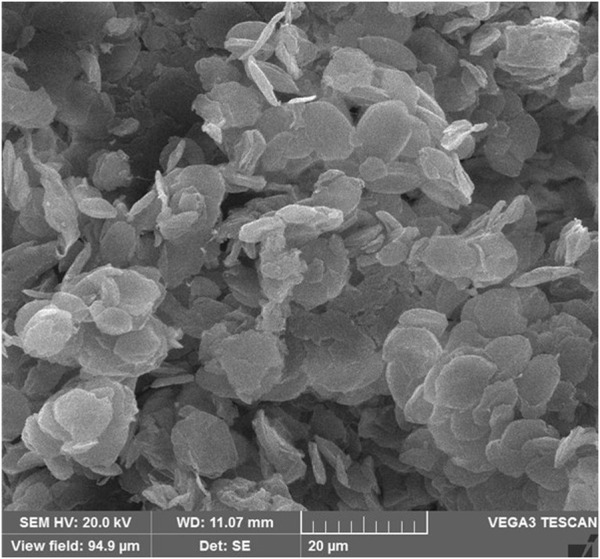
SEM image of the recovered PE/IL-WO_4_^=^ catalyst.

The SEM image of the recovered catalyst also showed no significant change in the catalyst morphology after seven recovery times confirming the high stability of the structure of the designed material during the applied conditions (Figure 9).

**FIGURE 10:**
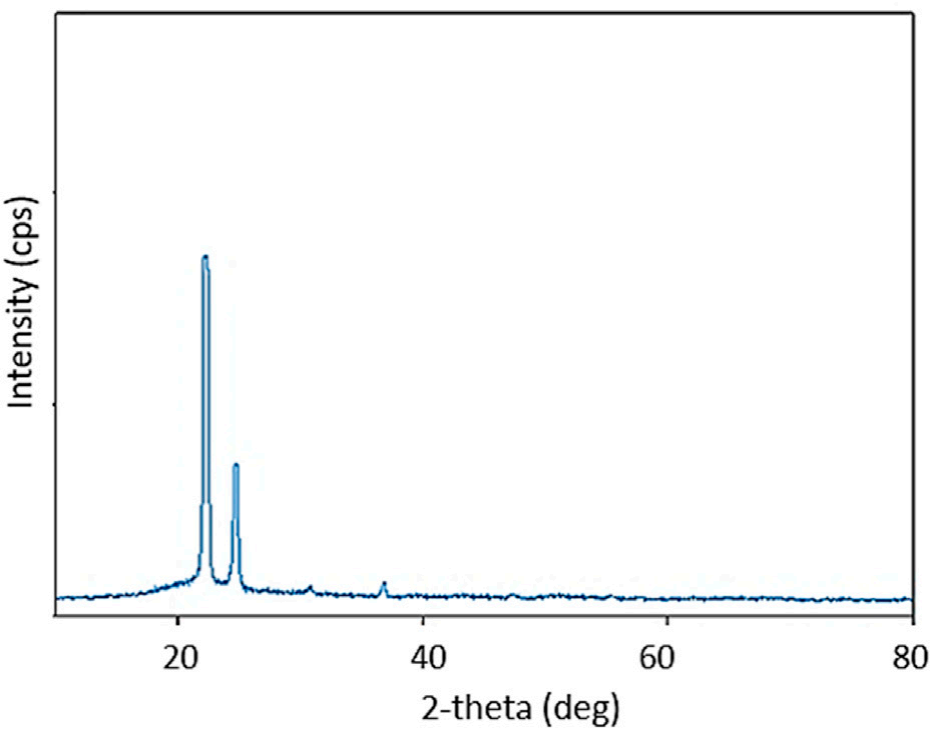
PXRD pattern of the recovered PE/IL-WO_4_^=^ catalyst.

The PXRD of the recovered PE/IL-WO_4_^=^ also showed four peaks at 2θ = 22.1°, 24.7°, 30.8°, and 36.9°, which are in good agreement with the PXRD pattern of the fresh catalyst. This analysis also confirms the high stability of the crystalline structure of PE after seven times of recovery and reuse (Figure 10).

In the subsequent study, a leaching test was conducted under applied conditions. To do this, once the reaction had reached approximately 50% completion, the PE/IL-WO_4_^=^ catalyst was separated, and the progress of the filtrate was monitored. After 120 min, no progress in the reaction was observed. Moreover, the atomic absorption analysis showed that the amount of W in the aforementioned filtrate is lower than 1 ppm. These results confirm no leaching and high stability of supported W sites and also the heterogeneous nature of the designed catalyst.

**FIGURE 11:**
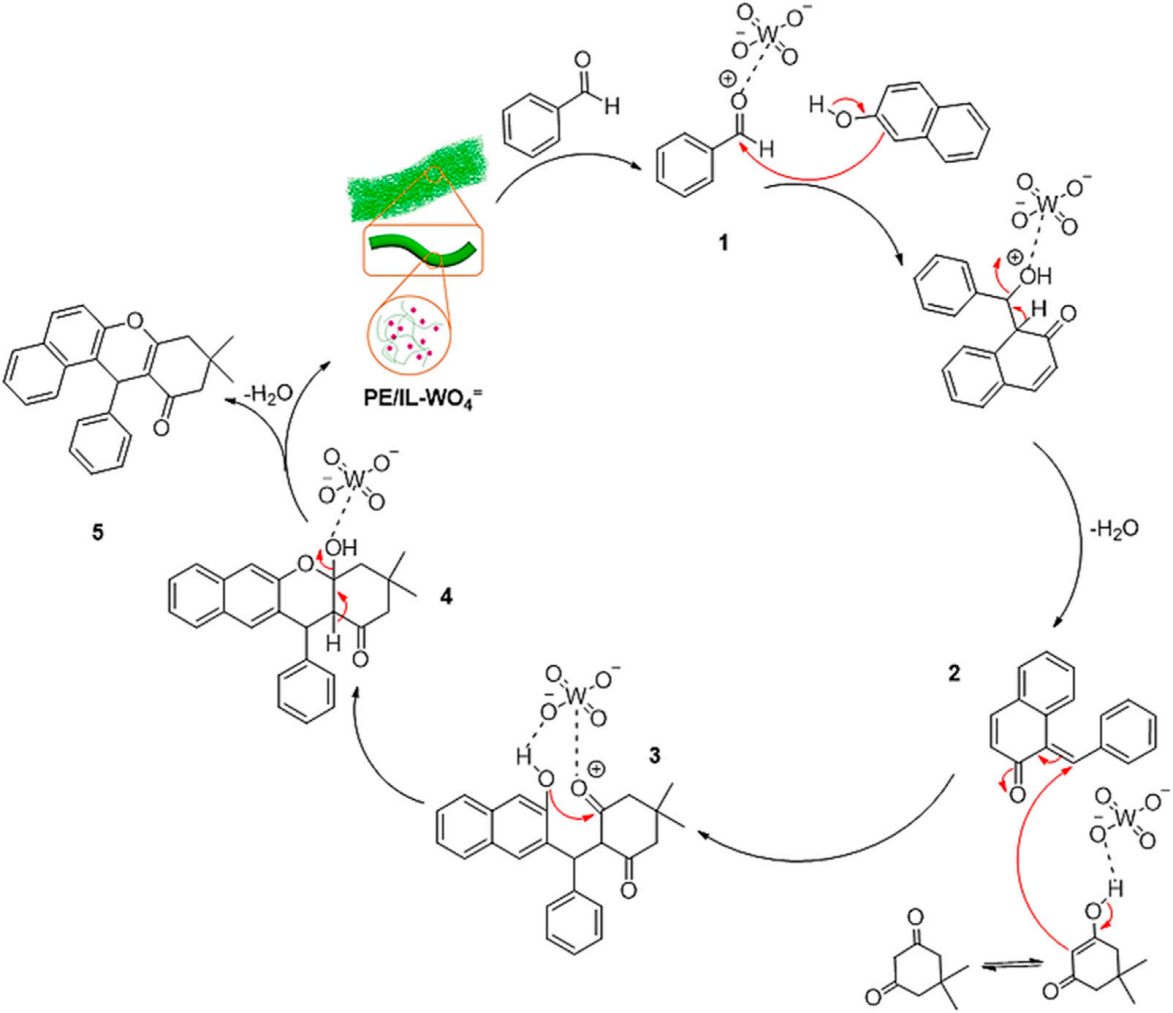
A plausible mechanism for the synthesis of tetrahydrobenzo [a]xanthen-11-ones.

A plausible mechanism for the synthesis of tetrahydrobenzo[a]xanthen-11-ones using the PE/IL-WO_4_^=^ catalyst is outlined in Figure 11. At the first step, a Knoevenagel condensation between W-activated aldehyde (1) and 2-naphthol gives intermediate 2. Then, intermediate 3 is formed via Michael-type addition between intermediate 2 and the enol form of dimedone. Finally, intermediate 3 undergoes an intramolecular cyclization followed by tautomerization in the presence of W-sites to give the desired product 5 with a high yield (Ardeshirfard and Elhamifar, 2023).

Next, the efficacy of the PE/IL-WO_4_^=^ catalyst in the synthesis of tetrahydrobenzo[a]xanthen-11-ones was compared to that of previous catalysts (Table 3). Overall, the results showed that the designed catalyst is better than the other catalysts in terms of temperature, catalyst loading, and number of recycling cycles. This better performance can be attributed to its highly lipophilic backbone, the incorporation of ILs into the material network to prevent leaching of the catalytic active site, and the bifunctional properties (both Lewis acidic and Lewis basic sites) of the supported tungstate. Moreover, PE, with its outstanding properties such as high thermal and chemical resistance, chemical inertness, and cost-effectiveness, provides distinct advantages over other supports.

**TABLE 3:** Comparative study of the performance of the present catalyst with that of previous catalysts.

Entry	Catalyst	Conditions (min)	Recovery times	Ref.
1	Fe_3_O_4_@nano-walnut shell/B^III^	Cat. 0.02 g, solvent-free, 80°C, 40	5	Abad et al. (2023)
2	HY zeolite	Cat. 20 mg, solvent-free, 80°C, 60	5	Rama et al. (2012)
3	HBF_4_/SiO_2_	Cat. 10 mol%, solvent-free, 80°C, 65	4	Zhang et al. (2009)
4	Cu/Fe_3_O_4_@APTMS-DFX^a^	Cat. 0.02 g, solvent-free, 120°C, 45	5	Sonei et al. (2019)
5	PE/IL-WO_4_^=^	Cat. 0.10 mol%, EtOH, reflux, 45	7	This work

^a^ 4-[3,5-Bis (2-hydroxyphenyl)-1,2,4-triazol-1-yl] benzoic acid (deferasirox).

## 4 Conclusion

In conclusion, a new composite consisting of high-density polyethylene (PE) and ionic liquid (IL)-WO_4_^=^ complex was synthesized, characterized and its catalytic application was investigated. The successful immobilization and great stability of the IL-WO_4_^=^ complex into/onto the polyethylene framework were confirmed through the utilization of FT-IR, TGA, SEM, PXRD, and EDX techniques. The tetrahydrobenzo[a]xanthen-11-one products were effectively prepared under green conditions using the PE/IL-WO_4_^=^ catalyst, resulting in high yields. With no appreciable decrease in efficiency, the PE/IL-WO_4_^=^ catalyst was recovered and reused at least seven times. The leaching test and also the atomic absorption analysis showed high stability and no leaching of catalytic active WO_4_^=^ species during reaction conditions. Moreover, the SEM and PXRD analyses confirmed the high durability of the structure of the designed catalyst under applied conditions. In light of these findings, future investigations on PE/IL-WO4^=^ are warranted to advance its applicability and understanding. As an example, the application of this catalyst in other catalytic processes such as coupling and oxidation reactions is underway in our laboratory. Moreover, both PE/IL and PE/IL-WO4^=^ can also be used as efficient adsorbents for the removal of pollutants from water.

## Data Availability

The original contributions presented in the study are included in the article/Supplementary material, further inquiries can be directed to the corresponding author.
